# Ability of *Rf5* and *Rf6* to Restore Fertility of Chinsurah Boro II-type Cytoplasmic Male Sterile *Oryza Sativa* (ssp. *Japonica*) Lines

**DOI:** 10.1186/s12284-017-0142-9

**Published:** 2017-01-21

**Authors:** Honggen Zhang, Jianlan Che, Yongshen Ge, Yan Pei, Lijia Zhang, Qiaoquan Liu, Minghong Gu, Shuzhu Tang

**Affiliations:** Jiangsu Key Laboratory of Crop Genetics and Physiology/Co-Innovation Center for Modern Production Technology of Grain Crops, Key Laboratory of Plant Functional Genomics of the Ministry of Education, College of Agriculture, Yangzhou University, Yangzhou, 225009 China

**Keywords:** *Japonica*, BT-type CMS, Fertility restorer gene (*Rf*), Gene mapping, Restoration ability

## Abstract

**Background:**

Three-line *Oryza sativa* (ssp. *japonica*) hybrids have been developed mainly using Chinsurah Boro II (BT)-type cytoplasmic male sterility (CMS). The *Rf1* gene restores the fertility of BT-type CMS lines, and is the only fertility restorer gene (*Rf*) that has been used to produce three-line *japonica* hybrids. Using more *Rf* genes to breed BT-type restorer lines may broaden the genetic diversity of the restorer lines, and represents a viable approach to improve the heterosis level of BT-type *japonica* hybrids.

**Results:**

We identified two major *Rf* genes from ‘93-11’ that are involved in restoring the fertility of BT-type CMS plants. These genes were identified from resequenced chromosome segment substitution lines derived from a cross between the *japonica* variety ‘Nipponbare’ and the *indica* variety ‘93-11’. Molecular mapping results revealed that these genes were *Rf5* and *Rf6*, which are the *Rf* genes that restore fertility to Honglian-type CMS lines. The BT-type F_1_ hybrids with either *Rf5* or *Rf6* exhibited normal seed setting rates, but F_1_ plants carrying *Rf6* showed more stable seed setting rates than those of plants carrying *Rf5* under heat-stress conditions. Furthermore, the seed setting rates of F_1_ hybrids carrying both *Rf5* and *Rf6* were more stable than that of F_1_ plants carrying only one *Rf* gene.

**Conclusion:**

*Rf6* is an important genetic resource for the breeding of BT-type *japonica* restorer lines. Our findings may be useful for breeders interested in developing BT-type *japonica* hybrids.

**Electronic supplementary material:**

The online version of this article (doi:10.1186/s12284-017-0142-9) contains supplementary material, which is available to authorized users.

## Background

Cytoplasmic male sterility (CMS) is caused by chimeric open reading frames in the mitochondrial genome, and is common in higher plants. It is a maternally inherited trait that results in an inability to produce functional pollen. Male sterility can be restored by the fertility restorer gene (*Rf*) in the nuclear genome (Hanson and Bentolila 2004). The CMS/*Rf* system has been widely used for hybrid seed production, and has helped to clarify the interactions between mitochondrial and nuclear genomes in plants (Chase 2007; Havey 2004).

The grain yields from three-line hybrid rice developed using the CMS/*Rf* system are 15–30% higher than those of inbred rice varieties (Fujimura et al. 1996; Yuan 1994). A CMS line, maintainer line, and a restorer line that carries the *Rf* gene are combined to develop three-line hybrids. For three-line hybrid rice, wild abortive (WA) and Honglian (HL) are the representative CMS types used for commercial *indica* hybrid seed production, and Chinsurah Boro II (BT) is the representative CMS type used for generating *japonica* hybrids (Chen and Liu 2014; Huang et al. 2014; Li et al. 2007; Yuan 1994). The BT-type cytoplasm has been identified from *Oryza sativa* ssp. *indica* Chinsurah Boro II and the BT-type CMS/*Rf* system is gametophytic. The BT-type CMS is caused by *orf79* in mitochondria (Wang et al. 2006), and the recovery of pollen fertility is regulated by *Rf1,* a fertility restorer gene on chromosome 10 (Shinjyo 1975; Wang et al. 2006). The *Rf1* gene has been cloned, and two alleles, *Rf1a* and *Rf1b*, have been identified at the *Rf1* locus (Kazama and Toriyama 2003; Akagi et al. 2004; Komori et al. 2004; Wang et al. 2006). *Rf1a* and *Rf1b* both encode pentatricopeptide-repeat containing proteins. RF1A can promote endonucleolytic cleavage of the *atp6–orf79* mRNA, whereas RF1B promotes degradation of *atp6–orf79* mRNA (Wang et al. 2006). Although progress has been made toward characterizing the mechanisms underlying the development of BT-type CMS and the restoration of fertility by *Rf* genes, only *Rf1* has been used to breed BT-type restorers. This has resulted in limited genetic diversity among BT-type restorers, and this is a major obstacle for the further development of BT-type *japonica* hybrids (Chen and Liu 2014). Thus, three-line *japonica* hybrids are not extensively cultivated in China (Deng et al. 2006). Also, F_1_ hybrids developed using the gametophytic CMS/*Rf* systems with multiple restorer gene loci can produce more than 50% normal pollen grains and exhibit good seed setting rates (Huang et al. 2012; Komori and Imaseki 2005), which may be useful for breeding BT-type *japonica* hybrids.

Several genetic loci related to the restoration of fertility in different types of CMS lines have been mapped in rice. *Rf3* and *Rf4* are two major fertility restorer genes for WA-type CMS, and have been mapped on chromosomes 1 and 10, respectively (Ahmadikhah and Karlov 2006; Tang et al. 2014; Zhang et al. 1997). *Rf5* and *Rf6*, which have been cloned and mapped on chromosomes 10 and 8, respectively, are the two major fertility restorer genes for HL-type CMS (Hu et al. 2012; Huang et al. 2000, 2012, 2015). *Rf17* (chromosome 4) and *Rf2* (chromosome 2) are the fertility restorer genes for Chinese wild rice-type CMS (Fujii and Toriyama 2009) and Lead rice-type CMS (Itabashi et al. 2011), respectively. Identifying new *Rf* genes and/or using previously reported *Rf* genes (except *Rf1*) to breed BT-type *japonica* fertility restorers may accelerate the development and application of BT-type *japonica* hybrids. This potentially represents an effective way to increase rice yields in China.

In this study, we used 57 chromosome segment substitution lines (CSSLs) derived from a cross between the *japonica* variety ‘Nipponbare’ (NIP; recipient parent) and the *indica* restorer ‘93-11’ (donor parent) as males to detect *Rf* genes for BT-type CMS. To identify possible *Rf* genes for BT-type CMS in ‘93-11’, a population of 127 CSSLs from this cross were reconstructed and resequenced. We then mapped the detected *Rf* genes and evaluated their ability to restore fertility to BT-type *japonica* CMS lines. Our results will be useful for breeding BT-type *japonica* restorer lines and for the development of three-line *japonica* hybrids.

## Results

### Construction of CSSLs and whole-genome resequencing

Between 2004 and 2010, a set of CSSLs was developed via crossing and backcrossing with the aid of 140 molecular markers, and the genotypes of 56 CSSLs (L1–L56) were determined using a high-throughput resequencing strategy. Based on the resequencing data, 152 substituted segments derived from ‘93-11’ were identified. These segments covered about 89.27% of the rice genome (Zhang et al. 2011). We observed that most lines contained five or more introgressed chromosome segments with many gaps. To obtain additional lines with a single segment and a high coverage rate, we reconstructed the CSSLs from the previously generated lines using backcrosses and marker-assisted selection (MAS) with 357 polymorphic molecular markers distributed relatively evenly across the 12 rice chromosomes. We ultimately obtained 351 CSSLs, and 127 lines were selected for resequencing. According to the resequencing results, the 127 CSSLs harbored 362 substituted segments derived from line ‘93-11’. The segments were 0.02–24.60 Mb long (average length: 5.39 Mb). For each CSSL, the number of substituted chromosomal segments varied from one to seven. Ninety-seven CSSLs contained two or fewer introgressed chromosomal segments, with 53 lines carrying only one segment. The total length of these substituted segments was 2023.8 Mb, which was 5.08 times the length of the rice genome. Furthermore, the CSSLs covered 94.93% of the ‘93-11’ genome (Table 1).

**Table 1:** Distribution of substituted segments from 127 CSSLs

Chromosome	Number of donor segment	Size of donor segment (Mb)	Density (%)
1	24	93.63	92.02
2	35	277.11	100
3	45	224.13	100
4	25	149.87	86.19
5	19	94.7	97.27
6	37	268.85	100
7	42	231.5	100
8	31	198.48	98.53
9	31	153.28	100
10	16	99.95	100
11	22	128.06	91.46
12	35	104.24	81.54
Total	362	2023.8	94.93

### Two major genes restore fertility of BT-type CMS lines

In the summer of 2011, the seed setting rate of 52 testcross F_1_ lines was analyzed. Six F_1_ hybrids derived from crosses between NIPA, a BT-type *japonica* CMS line with the nuclear background of NIP and L1, L18–20, L30, and L47 exhibited bagged and natural seed setting rates of 9.16–63.46% and 45.74–92.67%, respectively (Table 2). The other F_1_ hybrids showed bagged seed setting rates of zero and extremely low natural seed setting rates (i.e., < 5%). Therefore, we considered that these lines were all sterile and the natural seed setting rates of these lines were caused by outcrossing.

**Table 2:** Spikelet fertility of fertile F_1_ lines in testcross populations

Combination	Bagged seed setting rate (%) (mean ± SD)	Natural seed setting rate (%) (mean ± SD)
NIPA/L1	63.46 ± 19.24	82.05 ± 3.95
NIPA/L18	46.21 ± 11.08	92.67 ± 3.46
NIPA/L19	38.92 ± 15.15	85.18 ± 2.72
NIPA/L20	35.46 ± 11.91	92.12 ± 2.70
NIPA/L30	9.16 ± 4.73	45.74 ± 6.17
NIPA/L47	48.13 ± 7.95	89.42 ± 10.74

To detect quantitative trait loci (QTLs) associated with the fertility restoration of NIPA, we constructed a bin map based on 56 introgression lines, two parents, and the high-throughput resequencing data. A total of 366 bins were generated, with sizes of 0.01–11.92 Mb. The QTL analysis was conducted using the SARS program, and three QTLs for natural spikelet fertility were detected (Table 3). Among these QTLs, *qSF8-1* was located in bin 222 on chromosome 8, *qSF10-1* was located in bin 314 on chromosome 10, and *qSF12-1* was located in bin 349 on chromosome 12. Bin 222 was present in L1, L18, L19, and L20, while L30 and L47 harbored bin 314, and L47 harbored bin 349 (Table 3). The QTL mapping results revealed that bins 349 and 314 were present in L47. The chromosome interval related to bin 314 covered the *Rf1* locus (Akagi et al. 1996; Komori et al. 2004; Wang et al. 2006). Thus, we considered the possibility that *qSF12-1* does not exist.

**Table 3:** Mapping QTLs related to the fertility restoration of BT-type CMS lines

QTLs	Bin	Chr.	Interval	Size	Partial R-square	F
*qSF8-1*	222	8	1–688,039	688,038	0.6416	76.99
*qSF10-1*	314	10	16,949,055–18,486,880	153,7825	0.194	49.6
*qSF12-1*	349	12	4,816,707–6,620,947	1,804,240	0.2255	36.59

Because of the gametophytic manner in which *Rf* genes restore fertility in the BT-type CMS/*Rf* system, plants with the *rfrf* genotype were not present in the F_2_ populations. Therefore, most plants likely carried homozygous ‘93-11’ or heterozygous genotypes at the linkage marker loci if there was only one *Rf* gene that restored the fertility of BT-type CMS. Plants carrying the homozygous NIPA genotype were considered to be recombinants. In the spring of 2012, six F_2_ populations derived from the fertile F_1_ hybrids were planted, with each population consisting of 100 individuals. We developed five simple sequence repeat markers on chromosome 8 (1–688,039 bp) and two insertion/deletion markers on chromosome 10 (16,949,055–18,486,880 bp). The markers were used to detect polymorphisms between NIP and ‘93-11’. RM1019 and RM407 on chromosome 8 and STS10-16 and STS10-27 on chromosome 10 were determined to be polymorphic. RM407 was subsequently used to detect the genotypes of plants in the F_2_ populations derived from the following crosses: NIPA/L1, NIPA/L18, NIPA/L19, and NIPA/L20. All 400 analyzed plants carried homozygous ‘93-11’ or heterozygous genotypes. STS10-16 and STS10-27 were used to detect the genotypes of plants in the F_2_ populations derived from the NIPA/L30 and NIPA/L47 crosses, respectively. Almost all of the 200 examined plants exhibited homozygous ‘93-11’ or heterozygous genotypes. The exception was one plant that carried a homozygous NIPA genotype at STS10-27. These results indicated that all of L1, L18–20, L30, and L47 carried only one *Rf* gene, and that *qSF8-1* and *qSF10-1* were two major QTLs conferring the ability to restore fertility to BT-type CMS lines.

In the summer of 2014, natural spikelet fertility was assessed in 123 F_1_ lines derived from the cross between NIPA and the reconstructed CSSLs. Among the F_1_ lines, the populations generated from crosses between NIPA and four CSSLs (i.e., N91, N93, N116, and N119) exhibited natural seed setting rates of 90.95–94.51%, and the other lines were sterile. According to the high-throughput resequencing data, N91 and N93 carried a substituted segment covering *qSF8-1*, whereas N116 and N119 carried a substituted segment spanning *qSF10-1*. These results were confirmed by molecular detection with RM407, STS10-16, and STS10-27. Based on these results, we concluded that there were two non-allelic nuclear genes in ‘93-11’ that restored the fertility of BT-type CMS *japonica* lines.

### Molecular mapping of *qSF10-1* and *qSF8-1*

*qSF10-1* is located in a region that includes the *Rf1* locus, which contains *Rf1a*, or is tightly linked with *Rf1a* and *Rf1b* on chromosome 10 in BT-type *japonica* restorers (Akagi et al. 1996; Komori et al. 2004; Wang et al. 2006). *Rf5*, which is a dominant *Rf* gene associated with HL-type CMS in *indica* rice, is the same gene as *Rf1a* (Hu et al. 2012). To determine whether *qSF10-1* was *Rf1a* (*Rf5*), we sequenced the *Rf1a* allele in ‘93-11’, L37, and L47. Comparison of the nucleotide sequences of *Rf1a* and *Rf5* with those previously cloned from different restorers revealed that the sequence of the *Rf1a* allele from ‘93-11’is the same as the sequences of the corresponding genes in IR8 (Akagi et al. 2004) and Miyang 23 (Hu et al. 2012). This result confirmed that *qSF10-1* is *Rf1a* (*Rf5*).

To clarify the precise genomic position of *qSF8-1*, we developed 51 markers in the region containing RM407, and 14 polymorphic markers were obtained for mapping (Additional file 1: Table S1). In the summer of 2012, two markers (i.e., RM1019 and RM22271) were used to screen recombinant individuals from more than 4000 plants in the F_2_ and F_3_ populations. We identified 23 and 17 recombinants using RM1019 and RM22271, respectively. Another four markers (i.e., STS8-4, STS8-23, RM407, and STS8-32) were used to genotype the 40 recombinants. Among these recombined plants, two were detected by RM407 and one was detected by STS8-32. Thus, based on the reference sequence of the NIP genome, *qSF8-1* was localized to an approximately 35.5-kb region between the markers RM407 and STS8-32 on the short arm of chromosome 8, within which an open reading frame (ORF) (Os08g0110200) encoding PPR-containing protein might be the candidate gene for *qSF8-1*. We sequenced this ORF from 93-11 to L18, respectively. The nucleotide sequences were identical between 93-11 and L18, and 93-11 contains 16 nucleotide substitutions and one insertions of 2 bp at position +2315 compared with that of NIP. During our mapping work, Huang et al. (2012) reported the mapping results of the *Rf6* gene, which is also from ‘93-11’, and restores the fertility of HL-type *indica* CMS lines. Our mapping study essentially produced the same results. Furthermore, the cloning of *Rf6* indicated that *Rf6* is the ORF of Os08g0110200 and is also capable of restoring the fertility of BT-type CMS plants (Huang et al. 2015). Therefore, we concluded that *qSF8-1* is *Rf6*.

### Ability of *Rf6* to restore fertility is stable and two non-allelic restorer genes enhance heat stress tolerance in F_1_ hybrids

Among the samples sown in the field on May 10 (S1) in Yangzhou, most of the testcross F_1_ lines from the CSSLs headed on August 6–9, similar to the heading dates of NIP plants. In 2011, of the six fertile testcross F_1_ lines, the NIPA/L30 F_1_ plants headed on August 24. The daily maximum temperature during the flowering period was 24.0–28.0 °C (Additional file 2: Figure S1a), which was lower than the temperature during the flowering periods for the five other testcross F_1_ lines. The NIPA/L30 F_1_ plants exhibited a low seed setting rate (Table 2). These results indicated that exposure to low temperatures may influence the seed setting rates of F_1_ plants. Considering the temperature fluctuations during the flowering period of the testcross F_1_ lines, two sowing dates were used in 2012 [May 10 (S1) and May 20 (S2)], and 2013 [May 10 (S1) and June 5 (S3)]. In 2012, the testcross F_1_ plants and the F_2_ plants with different genotypes headed on August 6 and August 9, respectively. They also exhibited normal spikelet fertility (i.e., > 80%), indicating that *Rf5* and *Rf6* were able to restore fertility to BT-type CMS lines (Table 4). In 2013, the F_1_ plants and F_3_ plants carrying different genotypes headed on August 6 and August 20, respectively. The highest temperature (i.e., 37.0 °C) was first recorded on August 6, and high daytime temperatures continued for more than 1 week (Additional file 2: Figure S1b). Among the plants that headed on August 6, the seed setting rates of plants carrying heterozygous genotypes were relatively low, while the plants carrying homozygous genotypes exhibited normal seed setting rates (Table 4). In contrast, the seed setting rate was normal for all plants that headed on August 20. These results implied that heat stress affects the ability of *Rf5* and *Rf6* to restore fertility to BT-type CMS plants.

**Table 4:** Fertility levels of plants carrying different genotypes in 2012 and 2013

Combination	Genotype	Seed setting rate (%) (mean ± SD)			
		2012S1	2012S2	2013S1	2013S3
NIPA/L18 F_1_	*Rf6rf6*	87.41 ± 2.30	86.63 ± 5.95	67.22 ± 10.02	–
NIPA/L47 F_1_	*Rf5rf5*	–	–	36.85 ± 13.12	86.31 ± 6.56
(NIPA/L18) F_2_//L47 F_1_	*Rf6rf6Rf5rf5*	–	–	75.10 ± 12.91	–
NIPA/L18 F_2-4_	*Rf6Rf6*	90.12 ± 4.24	88.46 ± 3.64	82.46 ± 3.87	89.77 ± 5.17
	*Rf6rf6*	87.78 ± 6.89	87.32 ± 7.16	–	85.36 ± 5.39
NIPA/L47 F_2-4_	*Rf5Rf5*	86.89 ± 4.40	88.12 ± 3.52	82.77 ± 5.37	–
	*Rf5rf5*	85.45 ± 3.02	85.01 ± 5.88	41.37 ± 8.67	–

–: missed genotype

S1, S2, and S3 means the sowing dates of May 10, May 20 and June 5, respectively

**Fig. 1:**
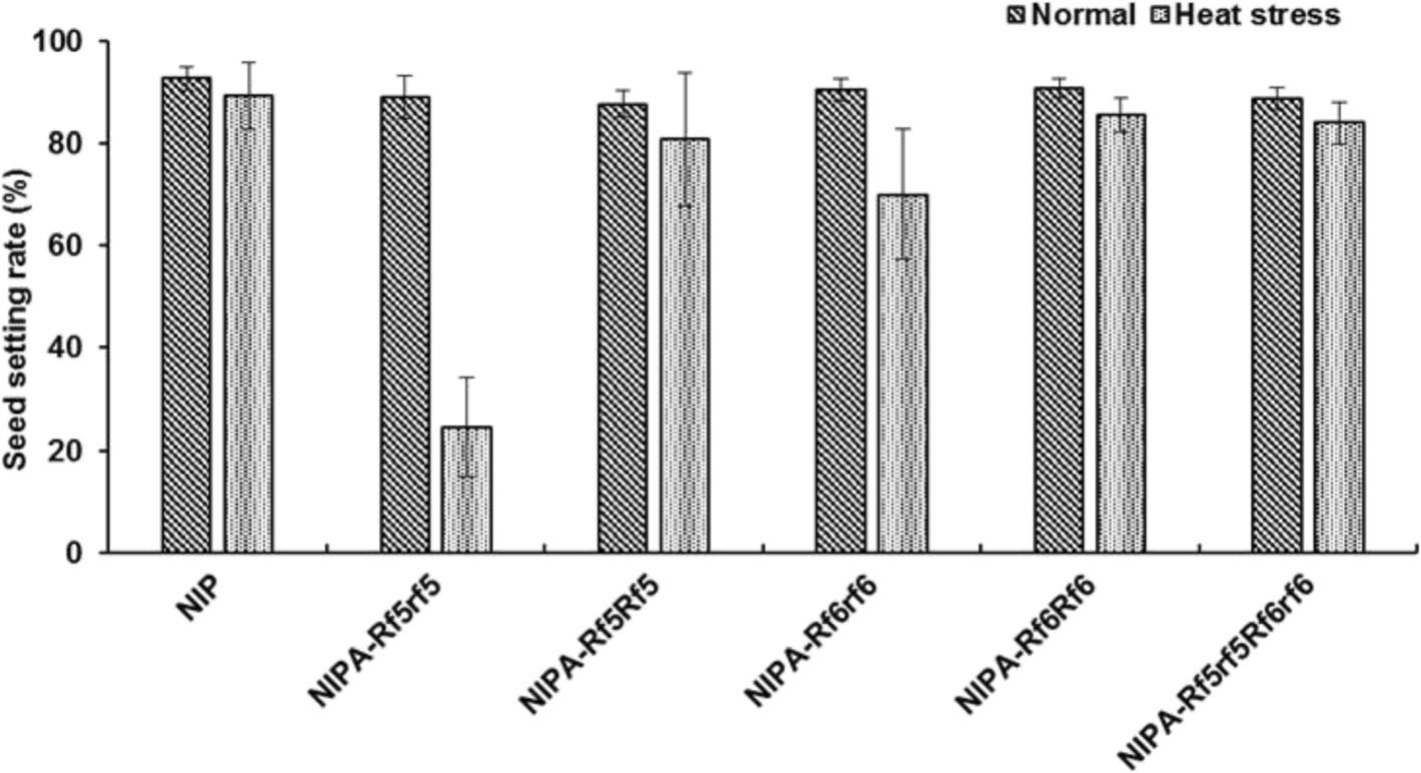
Effects of high temperatureson seed setting rates of plants harboring different genotypes. *Error bars* of seed setting rate is the mean value ± SD (*n* = 10). The heat stress is treated with the maximum temperatures of 37 °C

To further evaluate the ability of F_1_ hybrids with one or two *Rf* genes to adapt to heat stress, we developed the following three F_1_ populations: NIPA/L18 (*Rf6rf6*), NIPA/L47 (*Rf5rf5*), and (NIPA/L18) F_2_//L47 (*Rf5rf5Rf6rf6*). We also selected the plants harboring homozygous genotypes in the NIPA/L18 and NIPA/L47 F_4_ populations, which were exposed to high temperatures (25 °C nights, and maximum daytime temperatures of 37 °C) in 2014. These plants headed on August 6 when grown in the field, and on August 1 when grown in the greenhouse. Compared with the untreated plants, the F_1_ hybrids with only one *Rf* gene exhibited poorer seed setting rates, and the seed setting rate of plants with *Rf6* was higher than that of plants with *Rf5* (Fig. 1). In contrast, F_1_ plants carrying two restorer genes and plants with homozygous genotypes at *Rf* loci exhibited normal seed setting rates (Fig. 1). These results revealed that the presence of two non-allelic restorer genes in F_1_ hybrids increased the stability of their seed setting rate during exposure to environmental stresses such as high temperature.

## Discussion

Three-line *japonica* hybrids developed mainly with BT-type CMS lines have contributed greatly to rice production in China (Huang et al. 2014; Li et al. 2007). The BT-type CMS lines can be developed by nuclear substitution using *japonica* varieties from Japan and China as the recurrent paternal parent in backcrosses. Most BT-type *japonica* restorers contain only the *Rf1* locus (Akagi et al. 1996; Chen and Liu 2014; Huang et al. 2014; Komori et al. 2004; Wang et al. 2006), which was first transferred from *indica* varieties cultivated in Southeast Asia (Li et al. 2007). The F_1_ hybrids derived from BT-type *japonica* restorers exhibit a normal seed setting rate, with 50% of the produced pollen grains being fertile. Because BT-type *japonica* restorers contain only *Rf1*, the genetic diversity of restorers is relatively low. Additionally, the systematic use of heterosis for breeding BT-type *japonica* hybrid rice has been limited. Therefore, exploiting other *Rf* genes may be an effective way to increase the genetic diversity of BT-type restorers, which will likely enhance the development of *japonica* hybrids in China.

In this study, two *Rf* genes (i.e., *qSF8-1* and *qSF10-1*) involved in restoring the fertility of NIPA plants were identified from ‘93-11’, which is an elite *indica* fertility restorer for HL-type CMS. *Rf5* and *Rf6,* the two non-allelic fertility restorer genes in ‘93-11’ associated with HL-type CMS, have been mapped and cloned (Huang et al. 2012, 2015)*. Rf5* and *Rf1a* are the same gene, and *Rf6* is also capable of restoring the fertility of BT-type CMS plants (Huang et al. 2015). We mapped *qSF10-1* to a region on chromosome 10 containing *Rf5* (*Rf1a*) and mapped *qSF8-1* to a region on chromosome 8 containing *Rf6*. The ‘93-11’ *Rf5* and *Rf6* allele were sequenced. The sequencing results confirmed that *qSF10-1* is *Rf5* (*Rf1a*) and *qSF8-1* is *Rf6.* Therefore, the two ‘93-11’ *Rf* genes associated with BT-type CMS are *Rf5* and *Rf6*. Indeed, rice breeding experiments have revealed similarities in the restoration and maintenance relationship between BT-type CMS and HL-type CMS (Li et al. 2007; Zhang et al. 2016), consistent with the mapping results of this study.

The *Rf5* and *Rf6* genes exhibit similar abilities to restore the fertility of HL-type *indica* CMS lines (Huang et al. 2012). We observed that only a major gene (i.e., *Rf5* or *Rf6*) can restore normal fertility to BT-type CMS lines. However, this study revealed for the first time that the ability to restore fertility to BT-type *japonica* CMS lines is more stable with *Rf6* than with *Rf5* under heat-stress conditions. In the HL-type CMS/*Rf* system, *Rf6* and *Rf5* function via distinct mechanisms to rescue the sterility of HL-type CMS (Huang et al. 2012, 2015). Therefore, we hypothesize that the molecular mechanism underlying the fertility restoration of BT-type CMS plants differs between *Rf5* and *Rf6*, leading to the observed differences in the restorer activities of *Rf5* and *Rf6*. Additional studies are required to test this hypothesis.

In this study, F_2_ plants harboring *Rf5Rf5* or *Rf6Rf6* and plants harboring *Rf5rf5Rf6rf6* in the F_1_ population of a three-way cross exhibited the most stable fertility levels. These observations imply that breeding BT-type *japonica* restorers with multiple dominant *Rf* genes may increase the stability of seed setting in BT-type hybrid *japonica* rice. Therefore, using *Rf6* to restore fertility to BT-type CMS plants may facilitate the exploitation of heterosis in *japonica* breeding.

## Conclusion

We determined that *Rf5* and *Rf6* are the major *Rf* genes associated with BT-type CMS, and that the restorer activity of *Rf6* is more stable than that of *Rf5* under heat-stress conditions*.* The *Rf6* gene in ‘93-11’ may be an important resource for breeding BT-type *japonica* restorers to broaden the genetic diversity of BT-type rice. Also, combining *Rf6* with *Rf5* (*Rf1a*) in most pre-existing BT-type restorers may be an effective way to breed new BT-type restorers that exhibit stable seed setting abilities.

## Methods

### Plant materials

The donor parent used in this study was ‘93-11’, which is a typical *indica* cultivar and fertility restorer for BT-type and HL-type CMS. The recipient parent was NIP, which is a *japonica* cultivar and maintainer for BT-type CMS. The genomes of both parents have been sequenced. Between 2004 and 2010, a set of CSSLs derived from the cross between NIP and ‘93-11’ was developed by crossing and backcrossing with MAS, of which 56 lines were genotyped in 2010 using a resequencing method (Zhang et al. 2011). Based on the resequencing results, another 127 CSSLs from the same cross were further developed by backcrossing with MAS and then genotyped by resequencing (Additional file 3: Figure S2). In the spring of 2011 and 2014, these CSSLs were used as males for crosses with ‘NipponbareA’ (NIPA), which has the BT-type sterile cytoplasm with the nuclear background of NIP. We generated 52,123 F_1_ hybrids.

In 2011 and 2012, several F_2_ and F_3_ populations were generated from plants harboring the heterozygous genotype for *Rf* genes in the NIPA/CSSL testcross population. The corresponding progenies were used for the fine mapping of genes. To evaluate the ability of identified *Rf* genes to restore the fertility of BT-type CMS lines, we crossed NIPA with the CSSLs carrying various *Rf* genes. Plants harboring the homozygous or heterozygous genotypes for *Rf* genes in the F_2_–F_4_ populations were identified by MAS between 2012 and 2014. Plants harboring the homozygous genotype for *Rf* genes were crossed with each other in 2013 and 2014.

### Field experiment

All plant materials were sown on three dates [i.e., May 10 (S1), May 20 (S2), and June 5 (S3)] between 2011 and 2014 at the experimental field of Yangzhou University in Yangzhou, Jiangsu, China. Additionally, in the summer of 2014, plants carrying different genotypes for *Rf* genes were exposed to heat stress (25 °C nights, and maximum daytime temperatures of 37 °C) in a greenhouse for 10 days during the flowering period. Each field plot consisted of four rows separated by 25 cm, with each row consisting of five plants separated by 20 cm. Plants were grown using normal rice cultivation practices.

### Fertility scoring

In 2011, we analyzed the spikelet fertility in natural and bagged panicles of five plants from the CMS lines and each line in the testcross population. Between 2012 and 2014, only the spikelet fertility in natural panicles of five plants was assessed in the testcross F_1_ population, F_2_–F_4_ populations, and the three-way-cross F_1_ population. We counted the filled and unfilled grains of two panicles from one plant harvested at 20 days after flowering, and the spikelet fertility of one plant was measured as the average seed setting rate.

### Genotyping CSSLs by whole-genome resequencing

We used the high-throughput genotyping method developed by Huang et al. (2009). The CSSLs were genotyped based on single nucleotide polymorphisms (SNPs) generated from whole-genome resequencing as previously described (Xu et al. 2010). Briefly, at least 5 μg genomic DNA from each sample was randomly fragmented by sonication and electrophoretically size-fractionated. The DNA fragments approximately 500 bp long were then purified. Adapters were ligated to the purified fragments, which were then clustered and sequenced using the Illumina HiSeq 2000 system according to the manufacturer’s instructions (Illumina, San Diego, CA, USA). According to the published NIP genome sequence (http://rgp.dna.affrc.go.jp/IRGSP/Build4/build4.html), the detected SNPs were arranged along the chromosomes according to their physical locations. The genotypes of these CSSLs were evaluated by a group of consecutive SNPs using a sliding window approach, and different window sizes were used according to the SNP density of the CSSLs. The physical length of the substituted segments in each CSSL was estimated based on the resequencing results for each CSSL. The overlapping donor segments between or among different lines were used to divide the fragments into smaller segments (i.e., bins).

### Quantitative trait locus mapping and data analysis

Statistical analyses of the fertility of the plant materials and populations were conducted using the analysis of variance package in MATLAB (version 7.0). Commonly used bin mapping schemes were constructed and the contributions of the target bins to phenotypic variability were determined as described by Xu et al. (2010). The nomenclature of QTLs for natural spikelet fertility was according to the standard procedure described elsewhere (McCouch et al. 2002).

### DNA extraction, polymerase chain reaction, and sequencing

Genomic DNA was isolated from fresh leaves using cetyltrimethylammonium bromide (Rogers and Bendich 1985). Simple sequence repeat markers were identified using the Gramene database (http://www.gramene.org/). New insertion/deletion markers for the NIP (*japonica*) and ‘93-11’ (*indica*) genome sequences (http://www.ncbi.nlm.nih.gov/) were identified using the BLAST online tool. Primers were synthesized by Shanghai Sangon Inc. (Shanghai, China). Molecular marker analyses were conducted in 1× reaction buffer containing 0.1 mM dNTP, 1.0 U *Taq* polymerase, 0.2 μM primer, and 20 ng template DNA. The final volume was adjusted to 20 μL with ultra-pure water. The polymerase chain reaction (PCR) program was as follows: 94 °C for 4 min; 30 cycles of 94 °C for 45 s, 55 °C for 45 s, and 72 °C for 50 s; with final extension at 72 °C for 5 min. The amplification products were separated by 3% (*w/v*) agarose gel electrophoresis, detected with ethidium bromide, and visualized with a Gel Doc 1000 system (Bio-Rad Laboratories, Hercules, CA, USA).

According to the mapping results, the *Rf1a* allele and *Rf6* allele in ‘93-11’ were sequenced. The gene fragments were amplified by PCR using PrimeSTAR**®** GXL DNA polymerase (Takara, Dalian, China). The PCR products were purified using a TIANGEN PCR purification kit, and then ligated into the pEASY-Blunt cloning vector. The plasmid DNA was sequenced by GENEWIZ (Suzhou, China), and the correct DNA sequence was identified by comparing five plasmids. Sequence alignment was conducted with BLAST tools provided by the National Center for Biotechnology Information. The primers used to sequence the *Rf1a* and *Rf6* allele are listed in Additional file 1: Table S1.

### Accession number

Sequence data generated in this study have been deposited in the GenBank/EMBL database (accession numbers [KX517796, KY387609]).

## Additional files

Additional file 1: Table S1. Newly developed markers and primers used for gene mapping and sequencing. (DOCX 14 kb)
Additional file 2: Figure S1. Temperatures during August in 2011 (a) and 2013 (b). (DOCX 132 kb)
Additional file 3: Figure S2. Breeding strategy to produce chromosome segment substitution lines (CSSLs). Plant genotypes identified by marker-assisted selection (MAS) are indicated. (DOCX 126 kb)

## Abbreviations

BT: Chinsurah Boro II
CMS: Cytoplasmic male sterility
CSSL: Chromosome segment substitution lines
HL: Honglian
Nipponbare: NIP
NipponbareA: NIPA
PCR: Polymerase chain reaction
QTLs: Quantitative trait loci
*Rf*: Fertility restorer gene
