# Supplementary material for: Ability of Rf5 and Rf6 to Restore Fertility of Chinsurah Boro II-type Cytoplasmic Male Sterile Oryza Sativa (ssp. Japonica) Lines
Source: Rice (N Y). 2017 Jan 21;10:2. doi: 10.1186/s12284-017-0142-9 (PMC5253138; doi:10.1186/s12284-017-0142-9)
Supplement: Additional file 1: Table S1. — Newly developed markers and primers used for gene mapping and sequencing. (DOCX 14 kb) [file 12284_2017_142_MOESM1_ESM.docx]

Table S1. Newly developed markers and primers used for gene mapping and sequencing

| Primer | Forward Primer (5′-3′) | Reverse Primer (5′-3′) | Purpose |
| --- | --- | --- | --- |
| STS8-4 | CGTCGCCGATTGTTTCCT | CGGGCCGAGCTAGTTGAA | Mapping |
| STS8-21 | TTGTTGCTGGGTATGAAA | AAGAGTCCAAACGACGAG | Mapping |
| STS8-23 | ATAGAAACAAGAAATCAAAG | AACGGAAACAAAATACAC | Mapping |
| STS8-32 | AACAAAACAAAAGGACTC | GTGATAAGTATTATGGGACA | Mapping |
| *Rf1a*-1 | CACCTCTCCGTATAAGACAA | GGCATCAACAGCATAACATA | Sequencing |
| *Rf6-*1 | ATGTCGTCACGCCGTGTCC | TTACACAACTAATTCGATATAAGAAGAAG | Sequencing |
