# Supplementary figures and images for: Ability of Rf5 and Rf6 to Restore Fertility of Chinsurah Boro II-type Cytoplasmic Male Sterile Oryza Sativa (ssp. Japonica) Lines
Source: Rice (N Y). 2017 Jan 21;10:2. doi: 10.1186/s12284-017-0142-9 (PMC5253138; doi:10.1186/s12284-017-0142-9)

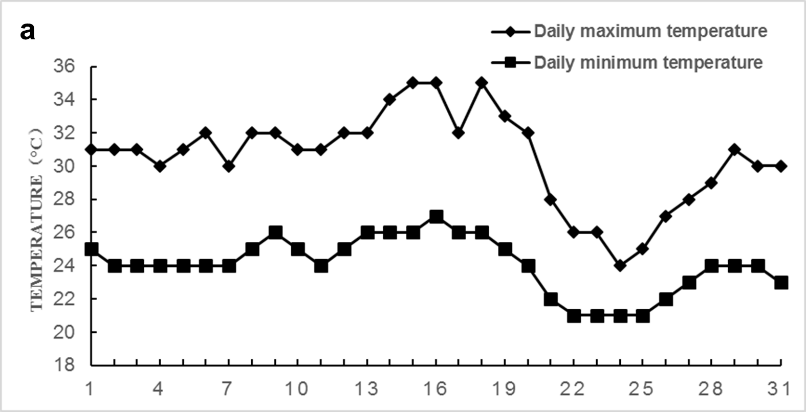


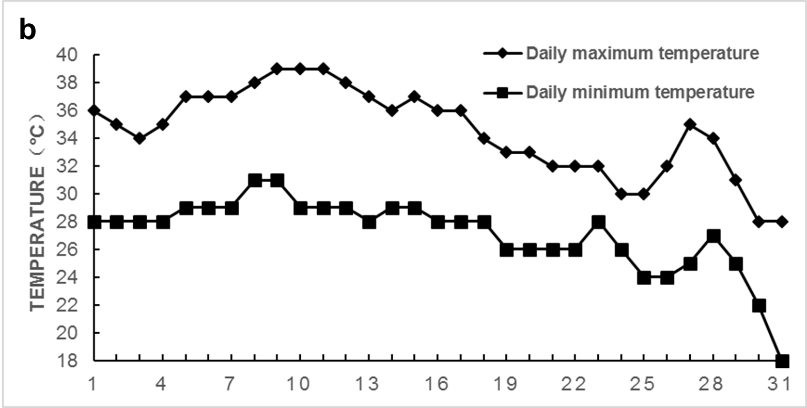


Figure S1 Temperatures during August in 2011(a) and 2013 (b)

Supplement: Additional file 2: Figure S1. — Temperatures during August in 2011 (a) and 2013 (b). (DOCX 132 kb) [file 12284_2017_142_MOESM2_ESM.docx]
