# Supplementary material for: Ability of Rf5 and Rf6 to Restore Fertility of Chinsurah Boro II-type Cytoplasmic Male Sterile Oryza Sativa (ssp. Japonica) Lines
Source: Rice (N Y). 2017 Jan 21;10:2. doi: 10.1186/s12284-017-0142-9 (PMC5253138; doi:10.1186/s12284-017-0142-9)
Supplement: Additional file 3: Figure S2. — Breeding strategy to produce chromosome segment substitution lines (CSSLs). Plant genotypes identified by marker-assisted selection (MAS) are indicated. (DOCX 126 kb) [file 12284_2017_142_MOESM3_ESM.docx]

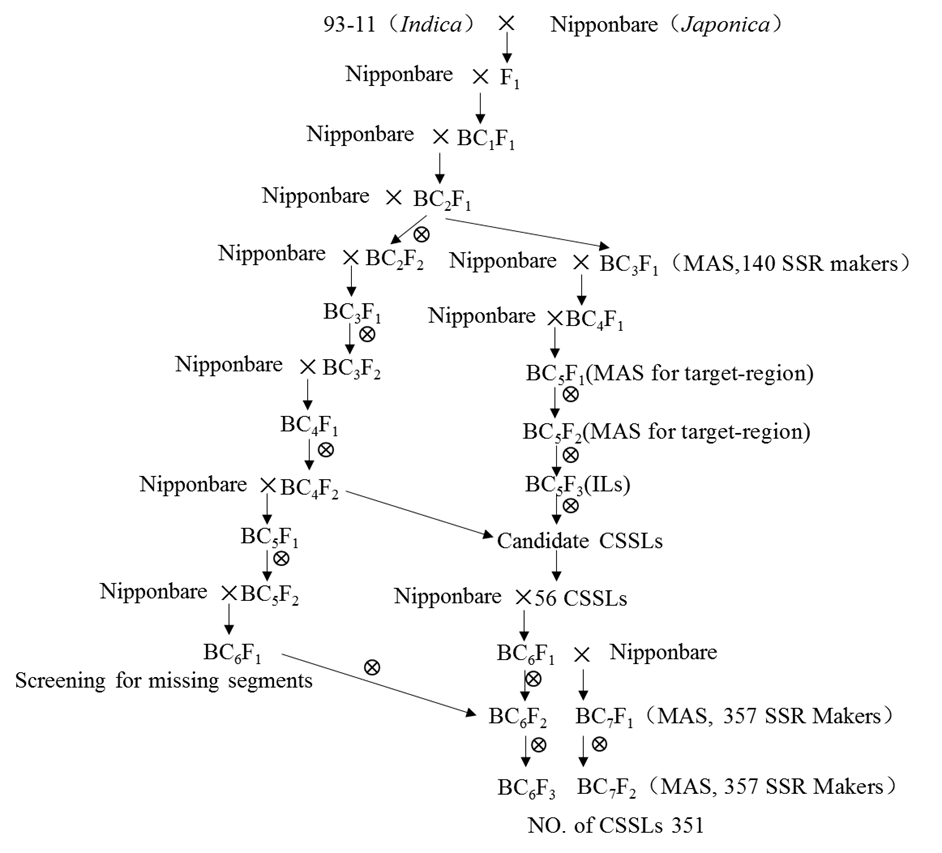


Figure S2 Breeding strategy to produce chromosome segment substitution lines (CSSLs). Plant genotypes identified by marker-assisted selection (MAS) are indicated.
